# Tnfaip6 Secreted by Bone Marrow–Derived Mesenchymal Stem Cells Attenuates TNBS-Induced Colitis by Modulating Follicular Helper T Cells and Follicular Regulatory T Cells Balance in Mice

**DOI:** 10.3389/fphar.2021.734040

**Published:** 2021-10-11

**Authors:** Guangli Gu, Xiaodan Lv, Gengfeng Liu, Ruizhi Zeng, Shiquan Li, Lan Chen, Zhaoliang Liang, Huiqin Wang, Fei Lu, Lingling Zhan, Xiaoping Lv

**Affiliations:** ^1^ Department of Gastroenterology, The First Affiliated Hospital of Guangxi Medical University, Nanning, China; ^2^ Department of Clinical Experimental Medicine, The First Affiliated Hospital of Guangxi Medical University, Nanning, China

**Keywords:** mesenchymal stem cells, inflammatory bowel disease, colitis, Tnfaip6, follicular helper T cells, follicular regulatory T cells

## Abstract

**Objective:** To investigate the immunological mechanism of bone marrow–derived mesenchymal stem cells (BM-MSCs) in inflammatory bowel disease (IBD).

**Methods:** Mice with 2,4,6-trinitrobenzene sulfonic acid (TNBS)–induced colitis were intraperitoneally injected with phosphate-buffered saline, BM-MSCs, BM-MSCs with tumor necrosis factor–induced protein 6 (Tnfaip6) knockdown mediated by RNA interference recombinant adenovirus, and BM-MSCs–infected with control adenovirus or recombinant mouse Tnfaip6. The disease activity index, weight loss, and histological scores were recorded. Serum levels of Tnfaip6 and pro- and anti-inflammatory cytokines, including interleukin (IL)-21, tumor necrosis factor-alpha (TNF-α), IL-10 were measured by enzyme-linked immunosorbent assay. The relative expression levels of these cytokines, B-cell lymphoma 6 (BCL-6) and fork-like transcription factor p3 (Foxp3) in the colon were determined by real-time quantitative PCR (RT-qPCR). BCL-6 and Foxp3 are the master regulators of follicular helper T cells (Tfh) and follicular regulatory T cells (Tfr), respectively. The infiltration of Tfh and Tfr in mesenteric lymph nodes (MLNs) and spleens was analyzed by flow cytometry.

**Results:** Compared to the normal control group, the expression levels of BCL-6 and IL-21 in the colon, Tfh infiltration, and ratios of Tfh/Tfr in the MLNs and spleen, and the serum concentrations of IL-21 and TNF-α increased significantly in the colitis model group (*p* < 0.05). Intraperitoneal injection of BM-MSCs or Tnfaip6 ameliorated weight loss and clinical and histological severity of colitis, downregulated the expression of BCL-6, IL-21, and TNF-α, upregulated the expression of Foxp3, IL-10, and Tnfaip6 (*p* < 0.05), increased Tfr and reduced the infiltration of Tfh in the MLNs and spleen, and downregulated the Tfh/Tfr ratio (*p* < 0.05). On the other hand, BM-MSCs lost the therapeutic effect and immune regulatory functions on Tfh and Tfr after Tnfaip6 knockdown.

**Conclusion:** Tfh increase in the inflamed colon, Tfh decrease and Tfr increase during the colitis remission phase, and the imbalance of the Tfh/Tfr ratio is closely related to the progression of IBD. Tnfaip6 secreted by BM-MSCs alleviates IBD by inhibiting Tfh differentiation, promoting Tfr differentiation, and improving the imbalance of Tfh/Tfr in mice.

## Introduction

Inflammatory bowel disease (IBD), including ulcerative colitis (UC) and Crohn's disease (CD), is characterized by an abnormal inflammatory response. In recent years, the global incidence and prevalence of IBD has been rising with highest prevalence in Europe and North America and rising incidence in newly industrialized countries in Africa, Asia, and South America (Ng et al., 2017; Jones et al., 2019; Xu et al., 2021). Studies have shown a greater predominance of CD in women than in men, but a greater predominance of UC in men than in women (Goodman et al., 2020). IBD severely affects the life quality of patients with a long course and recurrence. Current studies have shown that genetic susceptibility, immune response disorders, and environmental factors are involved in the pathogenesis of IBD. However, the precise etiology and pathogenesis of IBD are yet unclear. Since no curative treatments have been developed for IBD, it is important to study the pathogenesis of IBD and explore effective treatment methods.

Immune dysfunction is closely related to IBD. The differentiation of activated T cell subsets, including T helper (Th)-1 cells, Th9 cells, Th17 cells, and regulatory T cells (Treg), is unbalanced and a large number of pro-inflammatory cytokines such as tumor necrosis factor (TNF) α, interleukin (IL)-4, IL-5, IL-9, IL-13, and IL-33 are released, leading to tissue damage and impaired intestinal function (Gao et al., 2016; Friedrich et al., 2019). Follicular helper T cells (Tfh) and follicular regulator T cells (Tfr) are two newly found T helper cells in the germinal centers in recent years (Sage and Sharpe 2016; Vinuesa et al., 2016). Tfh is mainly regulated by transcription factor B-cell lymphoma 6 (BCL-6) and characterized by abundant production of IL-21 and highly expressed CD4, CXC chemokine receptor 5 (CXCR5), programmed cell death 1 protein (PD-1), and inducible co-stimulator (ICOS) and assists in the differentiation, proliferation, and secretion of antibodies of B cells (Dong 2021; Dong et al., 2021). IL-21 has an important positive regulatory effect on Tfh differentiation and acts directly on B cells (Dong et al., 2021). On the other hand, Tfr is mainly regulated by the fork-like transcription factor p3 (Foxp3) and characterized by abundant production of IL-10 (Canete et al., 2019), highly expressed CD4, CXCR5, and Foxp3, inhibition of inflammatory responses, and balancing of the immune-promoting effect of Tfh (Sage and Sharpe 2016). Besides, IL-10 inhibits the release of pro-inflammatory cytokines such as IL-21 and IL-17 and weakens B-cell help functions of Tfh (Cai et al., 2012; Yao et al., 2021). Recent studies have shown that the abnormal levels of Tfh/Tfr are closely related to the production of autoantibodies and the formation of germinal centers in various autoimmune diseases, such as systemic lupus erythematosus, rheumatoid arthritis, type 1 diabetes, and asthma (Xu et al., 2013; Gensous et al., 2017; Gensous et al., 2018; Liu et al., 2018; Gong et al., 2019). Our previous study also found that the number of Tfh cells in the intestinal germinal centers was increased in both UC and CD patients, whereas the number of Tfr cells reduced (Yang et al., 2020), suggesting that Tfh and Tfr are involved in the progression of IBD.

Mesenchymal stem cells (MSCs) have high proliferation and multidirectional differentiation capabilities, express immunosuppressive molecules and various growth factors, have strong immune regulatory functions, and maintain immune homeostasis. Clinical trials have proven that MSC transplantation is a promising treatment in IBD (Panes et al., 2018; Zhang et al., 2018; Cheng et al., 2019; Shi et al., 2019). Some studies have demonstrated that the immunomodulatory function of MSCs is mainly mediated by their secretory activity (Song et al., 2020), among which tumor necrosis factor–induced protein 6 (Tnfaip6) has a crucial role (Sala et al., 2015; Song et al., 2017). Tnfaip6 is the secreted protein product of tumor necrosis factor–stimulating gene-6 (*TSG-6*) with a molecular mass of about 35–38 kDa. It binds to a variety of ligands, such as matrix molecules, chemokines, and growth factors, and exerts immune regulation and tissue protection functions.

Tfh and Tfr are imbalanced in IBD. The therapeutic and immunomodulatory effects of MSCs on IBD depend on the secretion of Tnfaip6. Therefore, it could be speculated that MSCs alleviate IBD through the secretion of Tnfaip6 by improving Tfh/Tfr imbalance. In this study, we assessed the effects of bone marrow–derived (BM) MSCs on a 2,4,6-trinitrobenzene sulfonic acid (TNBS)–induced colitis mice model, together with the immunomodulatory effect on Tfh/Tfr, in order to identify the immunological mechanism of MSCs in the treatment of IBD.

## Materials and Methods

### Culture, the Subculture of Bone Marrow–Derived Mesenchymal Stem Cells, and Adenovirus Infection

BM-MSCs derived from Balb/c mouse and a complete medium set were purchased from Cyagen Biotechnologies (Suzhou, China). Cells were incubated in a complete medium containing 10% fetal bovine serum and 1% penicillin–streptomycin at 37°C in a 5% CO_2_ humidified incubator, following the manufacturer's instructions. At 80–90% confluency, the adherent cells were passaged after digestion with 0.25% (wt/v) trypsin/0.02% (wt/v) EDTA for 2–3 min. The culture medium was replaced every 2–3 days. BM-MSCs at passage 8 were used for subsequent transfection and transplantation experiments.

BM-MSCs were transfected with control adenovirus (GeneChem Inc., Shanghai, China) using the enhanced infection solution (GeneChem) or complete medium as the infection system for 8 h with multiplicity of infection (MOI) of 0, 50, 100, 200, and 400 to detect the best MOI. The non-transfected cells were taken as the naïve control. After 48 h of *in vitro* adenovirus infection, transfected and non-transfected BM-MSCs were harvested. To detect the infection rate and whether adenovirus affects the phenotype of MSCs, the cell morphology and growth state were observed, and the expression of green fluorescence protein (GFP) in mouse BM-MSCs and the cell surface molecules (CD31, CD45, and Sca-1) were detected by flow cytometry. Then, BM-MSCs were infected with mouse Tnfaip6 RNA interference (RNAi) recombinant adenovirus (GeneChem) at the best MOI. The expression level of *TSG-6* mRNA after transfection was determined by reverse transcription–quantitative polymerase chain reaction (RT-qPCR), and RNAi adenovirus with the highest interference efficiency at the mRNA level was screened out from three candidate targets. The target sequences of mouse Tnfaip6 RNAi recombinant adenoviruses were tgG​AAG​GTT​TAG​CCA​TCT​ATA, ccC​AAA​TGA​GTA​CGA​TGA​CAA, and ccC​GGC​TTC​AAT​ATT​TAT​ATT, labeled as virus A, B, and C, respectively. Finally, mouse BM-MSCs were infected with the selected RNAi adenovirus, and Tnfaip6 knockdown was confirmed by Western blotting.

To determine the phenotype after infection with control adenovirus, MSCs were suspended in Cell Staining Buffer (BioLegend, San Diego, California, USA) at a cell density of 10^6^ cells/100 μL/tube, stained with PE anti-Sca-1, FITC anti-CD31, and FITC anti-CD45 (BioLegend), at 4 °C for 15 min in the dark, and fixed with 500 µL of 4% paraformaldehyde. MSCs stained with isotype-matched control antibodies (PE anti-IgG2a and FITC anti-IgG2a; BioLegend) were taken as the control. Flow cytometry was conducted using a Navios system (Beckman Coulter, Miami, Florida, USA) and analyzed using Kaluza Analysis 2.0 and Navios software.

To detect *TSG-6* mRNA in MSCs, total RNA was extracted from BM-MSCs using a PrimeScript™ RT Reagent Kit with gDNA Eraser (TaKaRa Bio Inc., Shiga, Japan), according to manufacturer's instructions. First-strand cDNA was synthesized using 0.5 μg of total RNA. The cDNA samples were then amplified in the AriaMx Real-Time PCR system (Agilent Technologies, Palo Alto, California, United States) for 40 cycles (95°C for 5 s, 60°C for 20 s, and 72°C for 15 s) with specific oligonucleotide primers (TaKaRa, Dalian, China). Each sample was analyzed in triplicate, with *Tubulin* used as the housekeeping gene. The relative quantification of the target gene was determined using the ΔΔCT method. The primers used in RT-qPCR are listed in Table 1.

**TABLE 1:** List of genes screened by quantitative real-time PCR genes assessed using SYBR Green.

Gene		Primer sequences
*GAPDH*	Forward	5′-GGT​TGT​CTC​CTG​CGA​CTT​CA-3′
	Reverse	5′-TGG​TCC​AGG​GTT​TCT​TAC​TCC-3′
*IL-10*	Forward	5′-GCC​AGA​GCC​ACA​TGC​TCC​TA-3′
	Reverse	5′-GAT​AAG​GCT​TGG​CAA​CCC​AAG​TAA-3′
*IL-21*	Forward	5′-CTT​CGT​CAC​CTT​ATT​GAC​ATT​GTT​G-3′
	Reverse	5′-CCA​GGG​TTT​GAT​GGC​TTG​A-3′
*Foxp3*	Forward	5′-AGT​GCC​TGT​GTC​CTC​AAT​GGT​C-3′
	Reverse	5′-AGG​GCC​AGC​ATA​GGT​GCA​AG-3′
*BCL-6*	Forward	5′-TCC​ACA​GGA​AGC​AGG​AGA​GAG-3′
	Reverse	5′-GGT​CTG​GGG​TAG​GAA​ATG​GAG-3′
*TNF-α*	Forward	5′-CCC​TCA​CAC​TCA​GAT​CAT​CTT​CT-3′
	Reverse	5′-GCT​ACG​ACG​TGG​GCT​ACA​G-3′
*Tnfaip6*	Forward	5′-GGC​TGG​CAG​ATA​CAA​GCT​CA-3′
	Reverse	5′-TCA​AAT​TCA​CAT​ACG​GCC​TTG​G-3′

In order to determine the level of Tnfaip6, BM-MSCs were homogenized in radioimmunoprecipitation assay buffer (Solarbio, Beijing, China) mixed with protease inhibitor (Solarbio). Total protein concentration was quantified using a bicinchoninic acid protein assay kit (Solarbio), and the denatured protein extract was stored at −80°C until use. Proteins were resolved by sodium dodecyl sulfate–polyacrylamide gel electrophoresis (SDS-PAGE) and transferred to polyvinylidene fluoride membrane (Millipore, Boston, Massachusetts, United States). The membrane was blocked with 8% skim milk in Tris-buffered saline containing 0.1% Tween-20 (TBST) at room temperature for 3 h and probed with rabbit anti-mouse Tnfaip6 antibody (1:1,000, Cell Signaling, Boston, Massachusetts, United States) or tubulin antibody (1:1,000, Shanggong Inc., Shanghai, China) in TBST at 4°C for 12 h. Successively, the membranes were incubated with horseradish peroxidase (HRP)–conjugated goat anti-rabbit secondary antibodies (1:3,000, Abcam, Cambridge, United Kingdom) at 37°C for 1 h. Then, the immunoreactive bands were detected by enhanced chemical luminescence (Biosharp, Hefei, China).

### Animal Experiments and Colitis Induction

Balb/c mice (female, 8–9 weeks old, 19–21 g) were purchased from Charles River (Hangzhou, China), housed under standard conditions (controlled temperature 26–27°C, humidity 60–70%, and a 12-h light/dark cycle), and maintained for at least 1 week before the experiments began. The animal study was reviewed and approved by the Animal Care and Welfare Committee of Guangxi Medical University.

TNBS-induced colitis is characterized by T-cell–mediated immunity and widely used to explore immune regulation in IBD (Wirtz et al., 2017). This colitis model is more in line with the characteristics of transmural colitis of CD, which is characterized by a female preponderance (Goodman et al., 2020), so female mice were used in this study. Colitis was induced in mice by TNBS (Sigma-Aldrich, Saint Louis, Missouri, United States) as described previously (Wirtz et al., 2017). Briefly, the shaved skin was pre-sensitized with 150 μl of 1% (wt/v) TNBS–olive oil–acetone solution, and then 100 μl of 2.5% (wt/v) TNBS in 50% ethanol was injected into the colon of 24-h fasted mice 7 days after pre-sensitization. Mock-treated mice were administered olive oil–acetone solution and injected an equal volume of phosphate-buffered saline (PBS) through the anus as the normal control. Subsequently, the hair color, activity, weight change, stool consistency, and fecal occult blood test results were recorded daily. One day after enema (i.e., day 2), the mice were injected with a single intraperitoneal dose of BM-MSCs, BM-MSCs with or without Tnfaip6 knockdown infected by recombinant adenovirus (1×10^6^ cells in 100 μl PBS/mouse), or an equivalent volume of PBS as control, while each mouse in the Tnfaip6 group was injected intraperitoneally with 4 μg of recombinant mouse Tnfaip6 daily from days 2–5, as described in the literature (Sala et al., 2015). For this experiment, mice were randomly divided into six groups with 10 mice in each group: control, model (TNBS + PBS), MSCs (TNBS + BM-MSCs), Ad-MSCs (TNBS + BM-MSCs infected with control adenovirus), RNAi-MSCs (TNBS + MSCs infected with Tnfaip6 RNAi adenovirus), and Tnfaip6 (TNBS + Tnfaip6). On day 8, 150 μl of 4% (wt/v) chloral hydrate was injected intraperitoneally to anesthetize the mice. Next, blood samples were collected from the eye, and serum samples were obtained by centrifugation at 3,000 rpm at 4°C for 15 min. Then, the mice were sacrificed by cervical dislocation, and biological samples (colon tissue, mesenteric lymph nodes, and spleen) were obtained for further analysis. Serum samples were assessed by enzyme-linked immunosorbent assay (ELISA). Colon tissue was excised for histological assessment and RT-qPCR analysis. The MLNs and spleen were analyzed by flow cytometry to characterize Tfh and Tfr.

### Fecal Occult Blood Test

The fecal occult blood tests were performed on the mice from days 2–8 using commercial fecal occult blood test kits (dry chemical) (Ameritek, Jiaxing, China), according to the manufacturer's instructions.

### Evaluation of Colitis Severity and Histological Analysis

Weight loss and stool consistency were recorded daily during the entire experiment. Colon sections were fixed in 10% formaldehyde for 24 h, embedded in paraffin, and cut into 3-μm sections that were stained with hematoxylin and eosin and assessed for colonic damage microscopically. Histological examinations were performed in a blinded manner. The disease activity index (DAI) and inflammation-related histologic score were evaluated for each mouse. The DAI score was calculated based on the scores obtained for weight loss, stool consistency, and degree of intestinal bleeding: score = 0, less than 1% weight loss, normal stool, negative hemocult; score = 1, 1–5% weight loss, soft but still formed stool; score = 2, 6–10% weight loss, soft stool, positive hemocult; score = 3, 11–18% weight loss, very soft or wet stool, blood traces in stool visible; and score = 4, weight loss more than 18%, watery diarrhea, gross rectal bleeding. The criteria for histological score were graded as follows: 0 point = no evidence of inflammation; 1 point = low level of inflammation, with scattered infiltrating mononuclear cells (1–2 foci); 2 points = moderate inflammation, with multiple foci; 3 points = high level of inflammation, with increased vascular density and marked wall thickening; and 4 points = maximal severity of inflammation, with transmural leukocyte infiltration and loss of goblet cells (Wirtz et al., 2017).

### Quantitative Detection of mRNA Expression by Real-Time Quantitative PCR

RT-qPCR was used to assess the relative mRNA expression of molecules in the colon, including the Tfh main transcription factor *BCL-6*, the Tfr main transcription factor *Foxp3*, and related cytokines including *IL-10*, *IL-21*, and *TNF-α* with 1 μg total RNA as described above, and glyceraldehyde 3-phosphate dehydrogenase (*GAPDH*) was used as the housekeeping gene. Primers are listed in Table 1.

### Enzyme-Linked Immunosorbent Assay

The serum concentrations of Tnfaip6, IL-10, IL-21, and TNF-α of the mice were detected using commercial ELISA kits, according to the manufacturer's instructions. All ELISA kits were purchased from Shanghai MLbio Company in Shanghai, China, except the Tnfaip6 ELISA kit which was purchased from Signalway Antibody Company (College Park, Maryland, United States).

### Flow Cytometry Analysis

The spleen cells were collected in sterile ice-cold PBS and passed through a 200-µm cell strainer into a 15-ml tube by mechanical disruption using the plunger of a 5-ml syringe. The cells were pelleted by centrifugation at 1,500 rpm for 5 min and lysed with 4 ml red blood cell lysate buffer (Solarbio) for 5 min; the above filtration, collection, and centrifugation steps were repeated. The MLN cells were isolated by the same procedure except for the step of red blood cell lysis. To characterize Tfh and Tfr, the MLN and spleen cells (1×10^6^/tube) were stained with Pacific Blue™ anti-CD4, PE-Cy™7 anti-CXCR5, and APC anti-PD-1 (BD Biosciences, Franklin Lake, New Jersey, United States), at 4° for 30 min in the dark. Meanwhile, the cells were stained with matched isotype control antibodies (Pacific Blue™ anti-IgG2a, PE-Cy™7 anti-IgG2a, APC anti-IgG2, PE anti-IgG2a; BD Biosciences) and taken as the control. Then, the cells were stained with PE anti-Foxp3 at 4°C for 30 min after permeabilization and fixation using Foxp3/Transcription Factor Buffer Set (eBioscience, San Diego, California, United States). Finally, the cells were suspended in 500 µl of 4% paraformaldehyde, and the percentages of CD4^+^CXCR5^+^PD-1^+^ Tfh and CD4^+^CXCR5^+^Foxp3^+^ Tfr of the MLNs and spleen were detected by flow cytometry.

## Statistical Analyses

Data are presented as mean ± standard deviation. Statistical analysis was performed using one-way analysis of variance followed by Bonferroni test for data comparisons between three or more groups, except for grade data in DAI, and the histological score was evaluated using the Kruskal–Wallis test by GraphPad 5.0 software (GraphPad Software Inc., San Diego, California, United States). *p*-values < 0.05 were considered statistically significant.

## Results

### Detection of Adenovirus Transfection Efficiency

**FIGURE 1:**
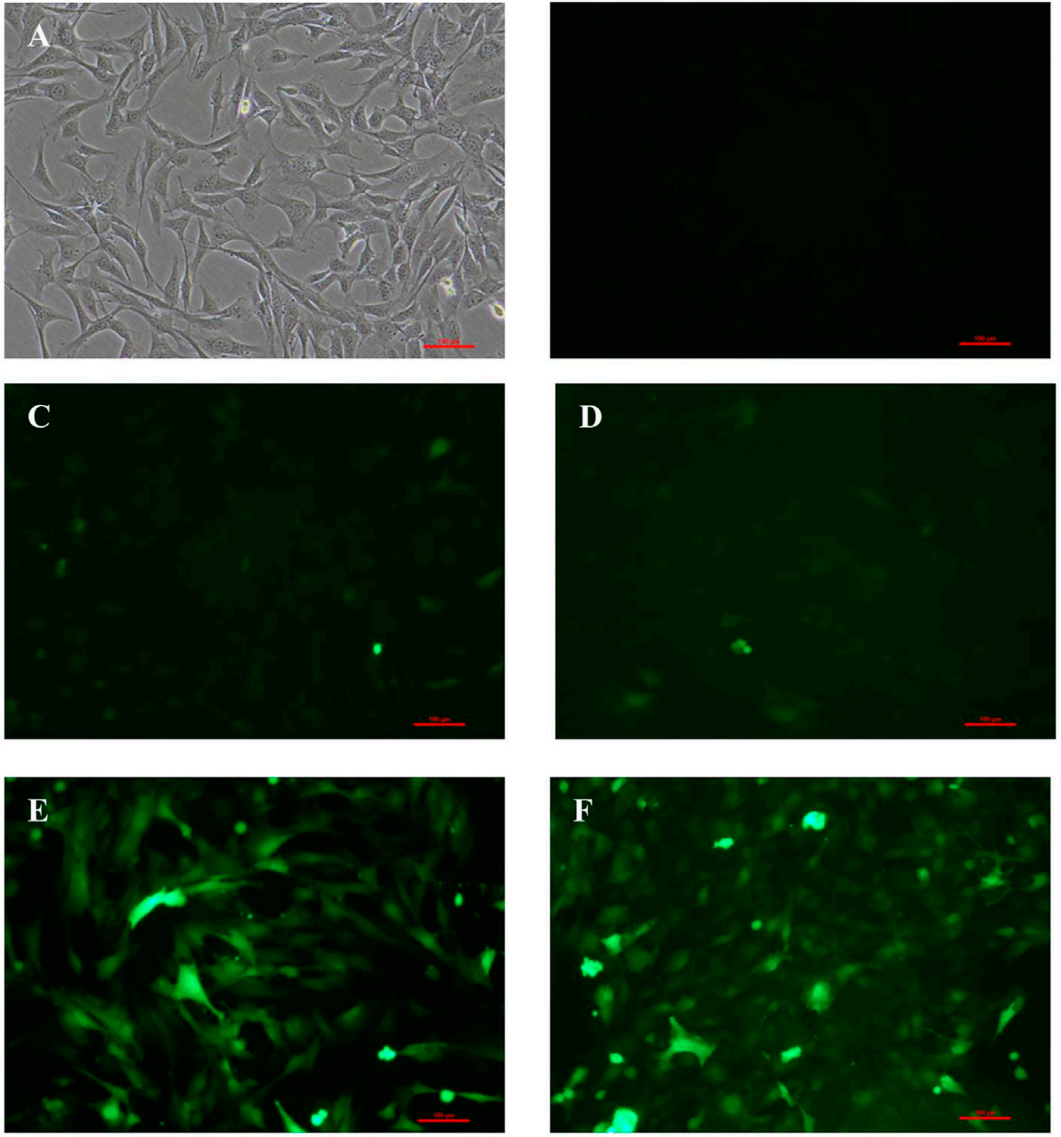
Expression of GFP in BM-MSCs 48 h after transfection at different levels of MOI. **(A)** Control BM-MSCs; **(B–F)** expression of GFP 48 h after BM-MSCs transfection by control adenovirus at different levels of MOI (MOI = 0, 50, 100, 200, and 400), ×100 magnification. *n* = 3 per group. Scale bar = 100 μm.

As shown in Figures 1B–E, 48 h later, BM-MSCs were found to be in good condition under the fluorescence microscope at MOI of 0, 50, 100, and 200. Slight green fluorescence was detected at the MOI of 50 and 100, while strong green fluorescence was observed at MOI of 200 and 400, and morphology retraction was detected in some of the cells at the MOI of 400 (Figure 1). These results indicated that the optimal MOI was 200.

**FIGURE 2:**
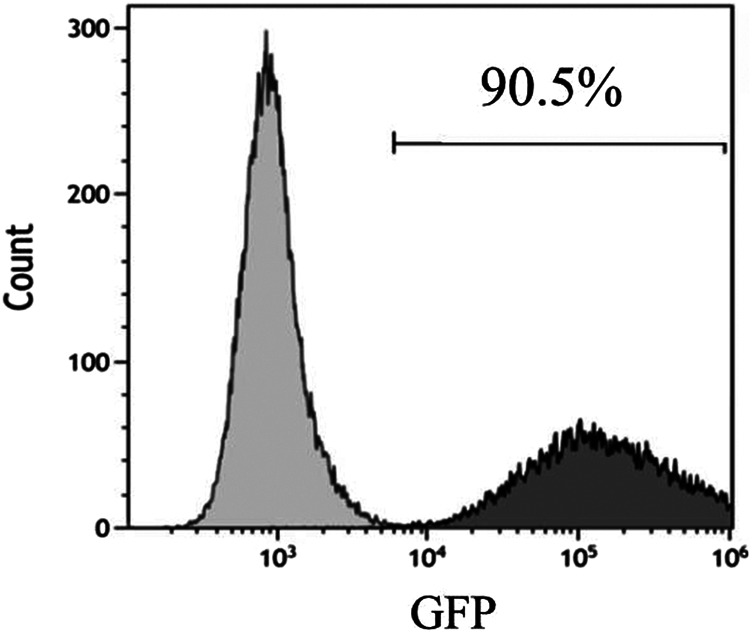
Infection rate of control adenovirus in MSCs detected by flow cytometry. MOI = 200. *n* = 3 per group.

**FIGURE 3:**
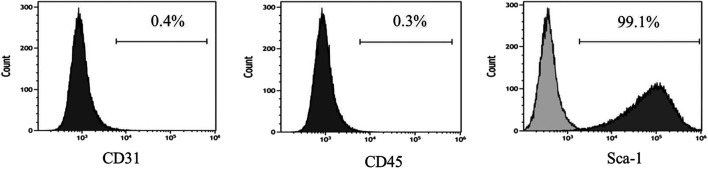
MSCs surface molecules detected by flow cytometry after infection by control adenovirus. *n* = 3 per group.

The adenovirus infection rate was about 90% (Figure 2). No obvious changes were observed in cell morphology and growth status postinfection. MSCs hardly expressed CD45 and CD31, and their positive rates were <0.5% after infection with control adenovirus. On the other hand, MSCs highly expressed Sca-1 with a positive rate >99% (Figure 3). The expression of CD31, CD45, and Sca-1 met the MSCs standards on the cell surface.

**FIGURE 4:**
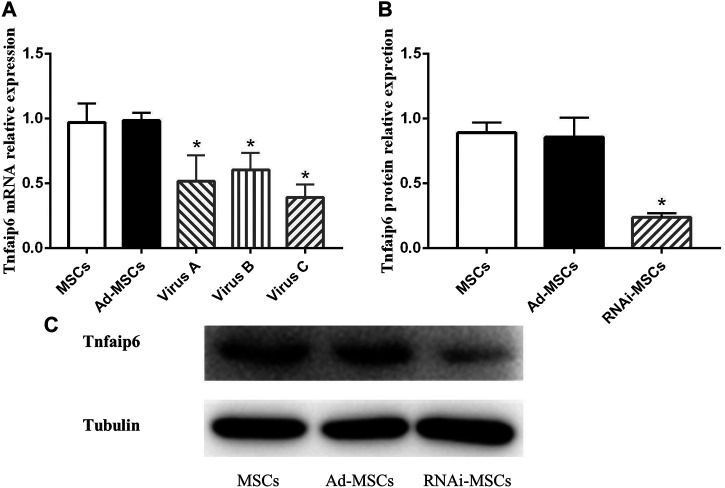
Tnfaip6 was knocked down in BM-MSCs after infection by recombinant RNAi adenovirus. **(A)** Relative expression levels of *TSG-6* mRNA detected by RT-qPCR in MSCs, Ad-MSCs, and RNAi-MSCs; **(B,C)** relative expression levels of Tnfaip6 protein detected by Western blotting in MSCs, Ad-MSCs, and target C RNAi-MSCs. MSCs: uninfected control BM-MSCs; Ad-MSCs: BM-MSCs infected with control adenovirus; and RNAi-MSCs: BM-MSCs infected with recombinant Tnfaip6 RNAi adenovirus C. ^*^ *p* < 0.05 compared to MSCs or Ad-MSCs group. *n* = 3 per group.

To determine the knockdown efficiency of transfected BM-MSCs, RT-qPCR and Western blotting were performed. As shown in Figure 4A, RNAi adenovirus C was confirmed with the highest interference efficiency at the mRNA level in the three candidate target sequences. The levels of Tnfaip6 in the transfected BM-MSCs were significantly lower than those measured from the naïve BM-MSCs and BM-MSCs infected with control adenovirus (Figures 4B,C).

These findings suggested that Tnfaip6 RNAi recombinant adenovirus has been successfully transfected into BM-MSCs, leading to a significant knockdown of Tnfaip6 but did not affect the phenotype of MSCs.

### Intraperitoneally Administered Bone Marrow–Derived Mesenchymal Stem Cells–Secreted Tnfaip6 Plays a Key Role in Attenuating TNBS-Induced colitis

**FIGURE 5:**
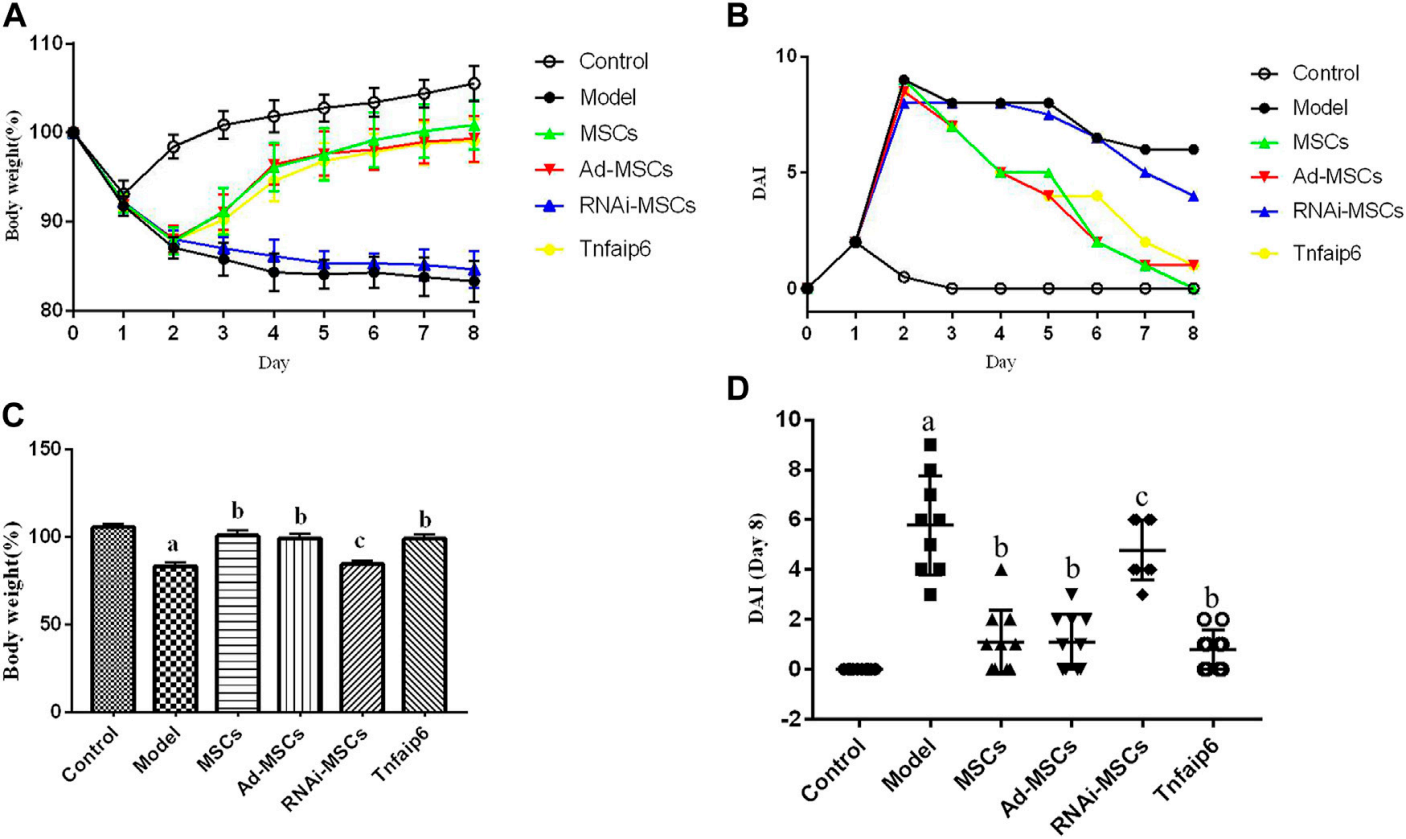
Intraperitoneal injection of MSCs or Tnfaip6 ameliorates TNBS-induced colitis in mice. **(A)** Changes in body weight; **(B)** changes of the DAI; **(C)** comparisons of body weight on day 8; and **(D)** comparisons of the disease activity index on day 8. ^a^ *p* < 0.05 compared to the control group. ^b^ *p* <0.05 compared to the model group. ^c^ *p* > 0.05 compared to the model group. *n* ≥ 9 per group.

**FIGURE 6:**
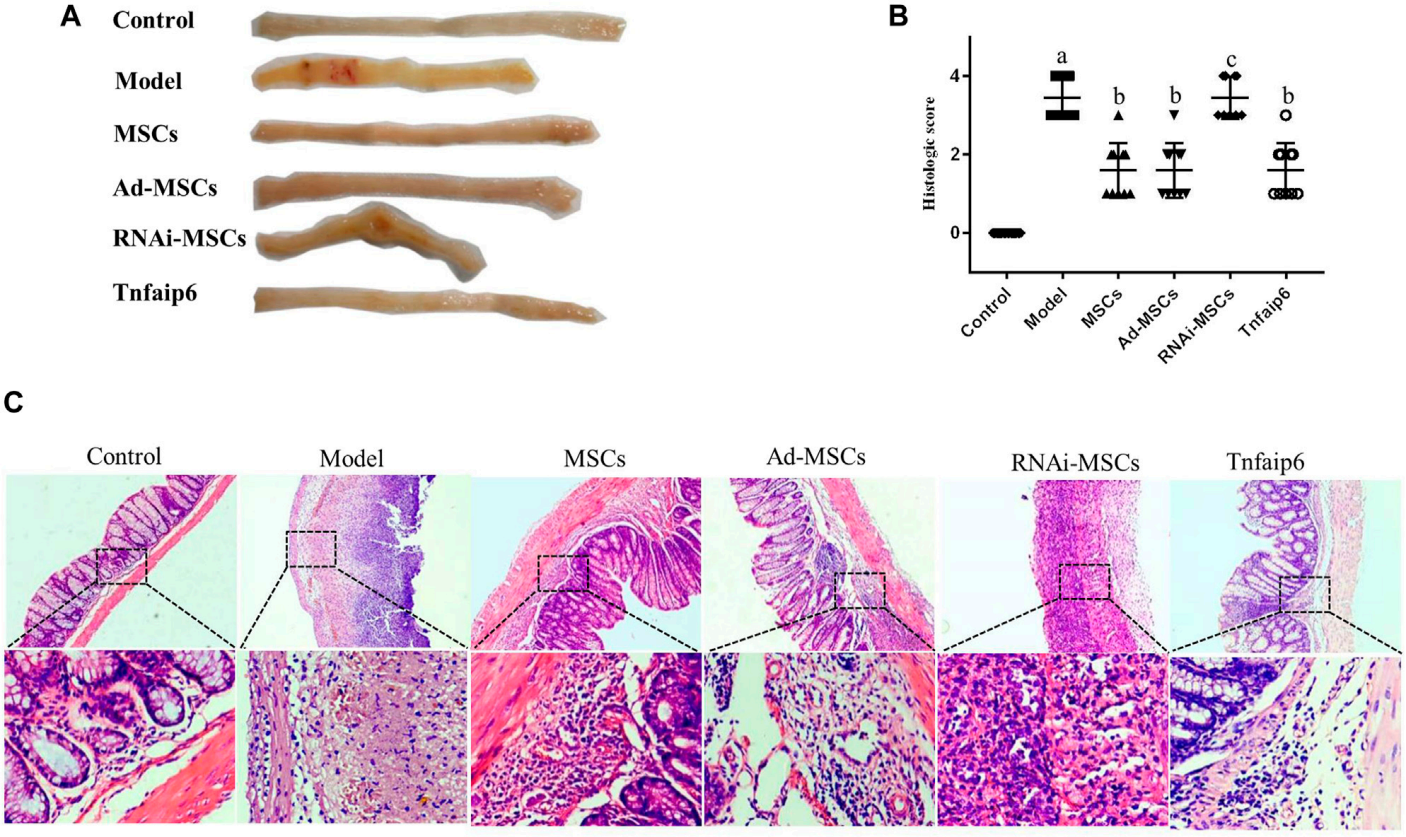
MSCs or Tnfaip6 ameliorates histological changes in TNBS-induced colitis in mice. **(A)** Colon tissue injury in each group: obvious ulcers and mucosal bleeding in the model and RNAi-MSCs groups, no visible lesions in other groups. **(B)** Inflammation-related histologic scores. **(C)** Representative HE-stained images of colon sections (×40 and ×200 magnification). Control group: normal colon; model and RNAi-MSCs group: infiltrating inflammatory cells, increased vascular density and marked wall thickening, loss of goblet cells, and penetrating ulcer; and MSCs, Ad-MSCs, and Tnfaip6 groups: intact intestinal wall with mucosal and/or submucosal inflammatory cell infiltration. ^a^ *p* < 0.05 compared to the control group. ^b^ *p* < 0.05 compared to the model group. ^c^ *p* > 0.05 compared to the model group. *n* ≥ 9 per group.

On days 6 and 7, one mouse died in the model and Tnfaip6 knockdown MSCs groups, and no mice died in the other groups before blood collection. On the following days of TNBS enema, mice in the model group gradually developed arched backs, lazy movements, reduced food and water intake, and roughened hair and reduced gloss, and soft, loose, mucous, watery, or even bloody stools. Fecal occult blood tests were positive, weight was gradually decreased, and DAI was significantly increased (Figures 5A,B). In the later stage of modeling, due to the body's self-healing tendency, the feces became thin and soft with mucus attached to the surface, the positive rate of the occult blood tests decreased, the weight of some mice stabilized or rebounded slightly, and the DAI decreased gradually (Figures 5A–D). On day 8, mice were sacrificed, and the histology of the colon was assessed. Some colitis mice had an intestinal obstruction and showed adhesion of intestinal tissue to peripheral tissues at the obstructed site. The histological examination revealed infiltrating inflammatory cells, increased vascular density and marked wall thickening, loss of goblet cells, and penetrating ulcer in TNBS-induced colitis mouse colons (Figures 6A–C).

After intraperitoneal injection of BM-MSCs, Ad-MSCs, or Tnfaip6, the weight loss and DAI score were all lower than those in the model group from day 4 (*p <* 0.05), and no significant differences were detected in the body weight loss and DAI among mice injected with BM-MSCs, Ad-BM-MSCs, or Tnfaip6 (*p* > 0.05) (Figures 5A–D). After the administration of MSCs, Ad-MSCs, or Tnfaip6, the intestinal obstruction and adhesion of intestinal tissue were significantly improved, no transmural ulcers were observed, glands were more orderly than those in the model group, inflammatory infiltrations were significantly reduced, and inflammation-related histologic score was lower than that of the model group (*p <* 0.05) (Figures 6A–C). However, after injected with RNAi-MSCs, no statistical differences were detected in the weight loss, DAI, and the inflammation-related histologic score compared to that of the model group (*p* > 0.05) (Figures 5, 6). The above results indicated that BM-MSC transplantation ameliorated TNBS-induced colitis in mice. The injection of recombinant Tnfaip6 achieved similar effects as BM-MSC transplantation (Figures 5, 6). BM-MSCs lost the therapeutic effect of colitis after Tnfaip6 was knocked down in the IBD mice model (Figures 5, 6).

### Bone Marrow–Derived Mesenchymal Stem Cells–Secreted Tnfaip6 Modulates the Immune Response in TNBS-Induced Colitis

**FIGURE 7:**
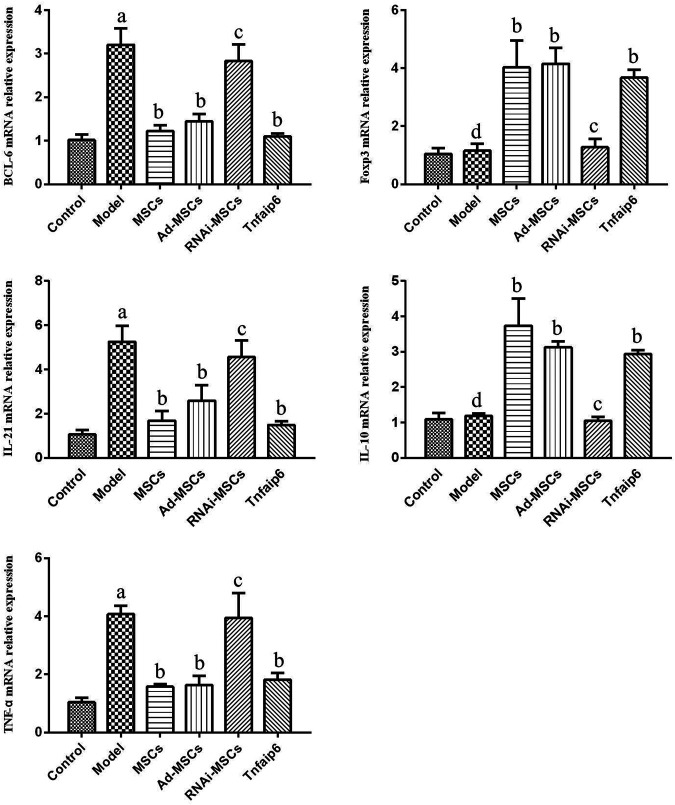
Relative mRNA expression levels of *BCL-6*, *Foxp3*, *IL-21*, *IL-10*, and *TNF*-*α* determined by RT-qPCR in the colon. ^a^ *p* < 0.05 compared to the control group. ^b^ *p* > 0.05 compared to the model group. ^c^ *p* > 0.05 compared to the model group. ^d^ *p* > 0.05 compared to the control group. *n* ≥ 9 per group.

**FIGURE 8:**
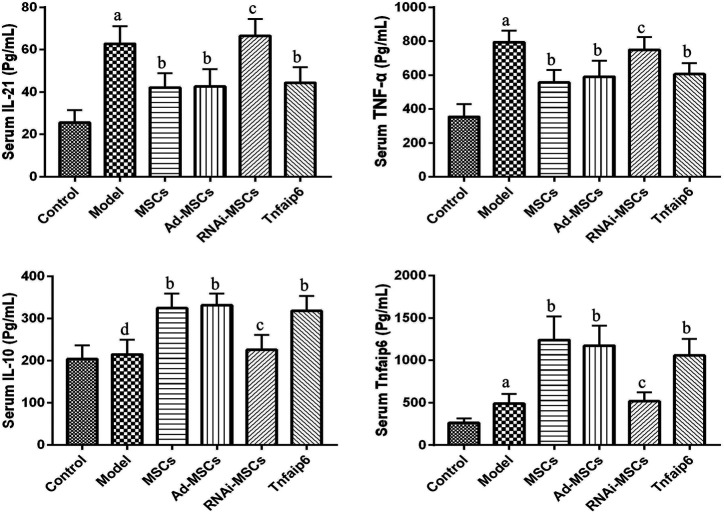
Serum concentrations of IL-21, TNF-α, IL-10, and Tnfaip6 in mice determined by ELISA. ^a^ *p* < 0.05 compared to the control group. ^b^ *p* < 0.05 compared to the model group. ^c^p > 0.05 compared to the model group. ^d^ *p* > 0.05 compared to the control group *n* ≥ 9 per group.

Next, we evaluated the effect of BM-MSCs on the modulation of inflammatory cytokines and molecules closely related to the differentiation and immune regulatory effects of Tfh and Tfr in TNBS-induced colitis, assessed the relative expression level of Tfh's main transcription factor *BCL-6* and Tfr's main transcription factor *Foxp3* mRNA by RT-qPCR in inflamed colons, and detected serum IL-21, TNF-α, IL-10, and Tnfaip6 concentrations. The relative expressions of *BCL-6*, *IL-21*, and *TNF-α* mRNA were higher in the inflamed colon of the model group compared to those of the control group (*p <* 0.05), accompanied by the same tends of serum IL-21, TNF-α, and Tnfaip6, while IL-10 and Foxp3 were slightly increased without statistical differences (*p* > 0.05) (Figures 7, 8). After intraperitoneal injection of BM-MSCs or Tnfaip6, the expression of BCL-6, TNF-α, and IL-21 were significantly decreased (*p <* 0.05) (Figures 7, 8), while expression of Foxp3, IL-10, and Tnfaip6 was significantly higher than those of the model group (*p* > 0.05) (Figures 7, 8). However, inhibition of Tnfaip6 significantly reduced the anti-inflammatory abilities of BM-MSCs to modulate BCL-6, Foxp3, TNF-α, IL-21, and IL-10 in the colon and/or serum (Figures 7, 8).

### Bone Marrow–Derived Mesenchymal Stem Cells–Secreted Tnfaip6 Modulates Tfh/Tfr in TNBS-Induced Colitis

**FIGURE 9:**
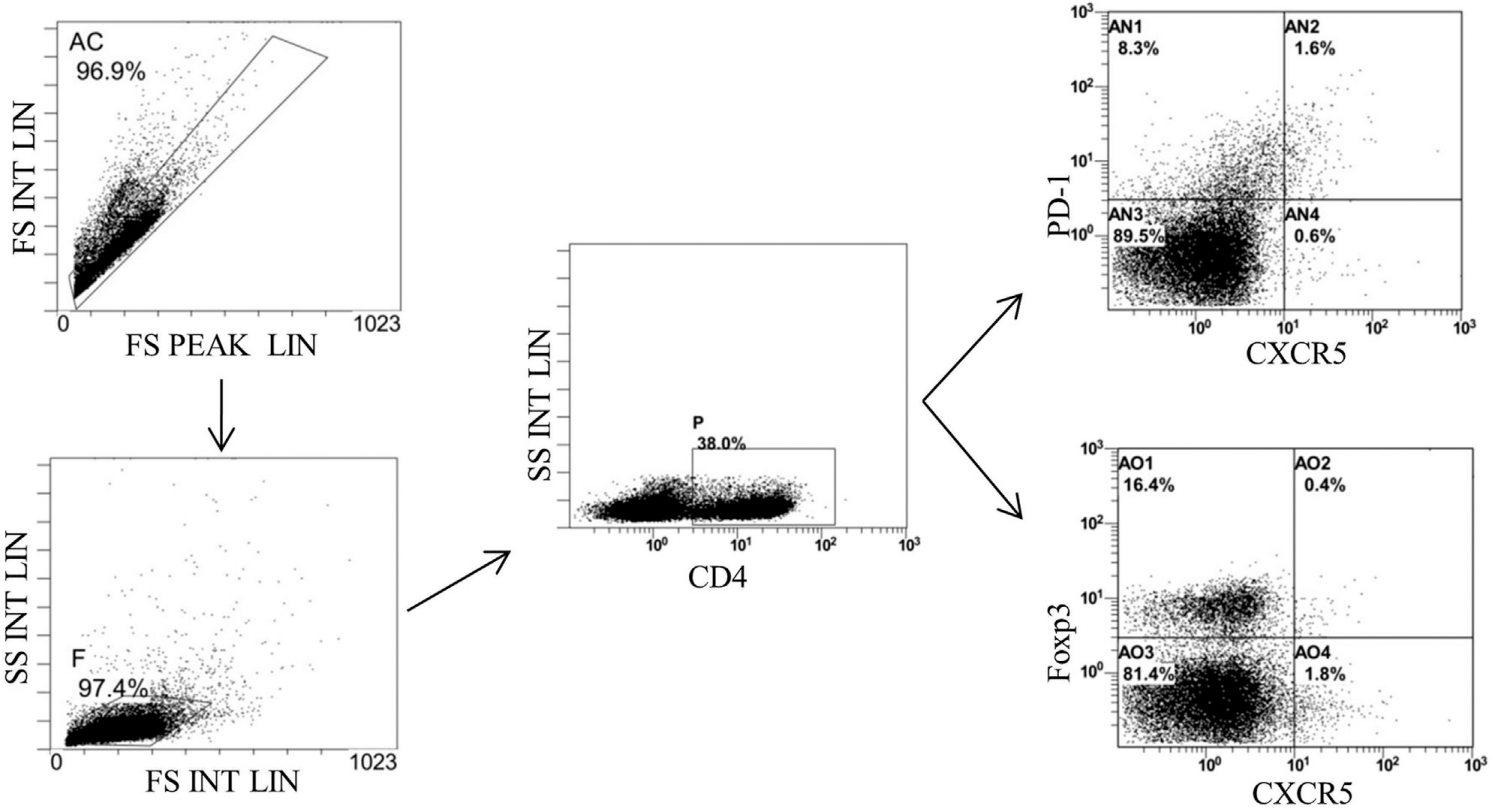
Gating strategy of Tfh and Tfr in the MLNs of mice detected by flow cytometry. Gate AC: nonadherent cells; gate F: lymphocytes; gate P: CD4^+^ cells; gate AN2: CD4^+^CXCR5^+^PD-1^*^ *T*th in CD4^+^ cells; gate A02: CD4^+^CXCR5^+^Foxp3^+^ Tfr in CD4^+^ cells; and MLNs: mesenteric lymph nodes.

**FIGURE 10:**
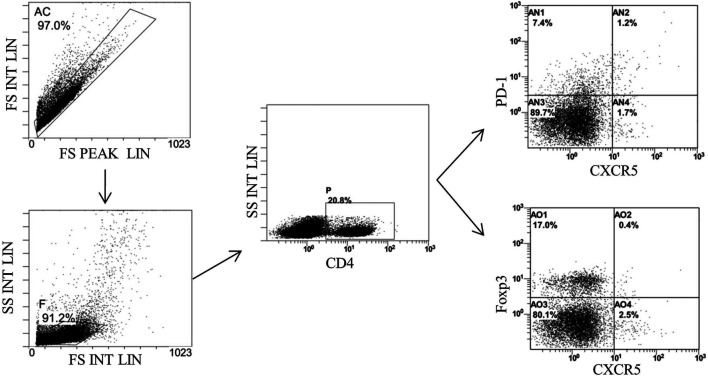
Gating strategy of Tfh and Tfr in the spleen of mice detected by flow cytometry. Gate AC: nonadherent cells; gate F: lymphocytes; gate P: CD4^+^ cells: gate AN2: CD4^+^CXCR5^+^PD-1^*^ Tth in CD4^+^ cells; and gate AO2: CD4^+^CXCR5^+^Foxp3^+^ Tfr in CD4^+^ cells.

**FIGURE 11:**
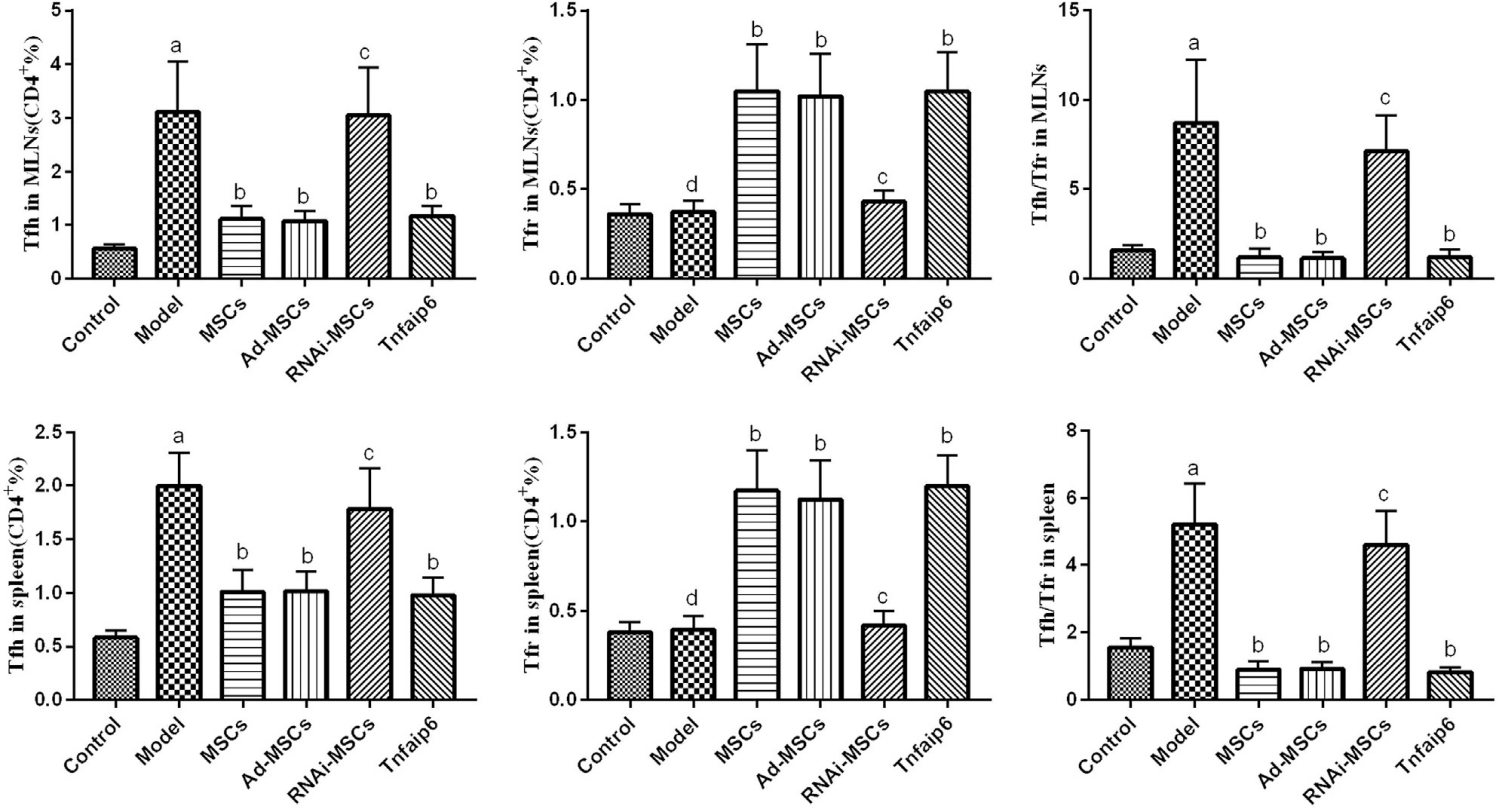
Comparisons of Tfh, Tfr, and Tfh/Tfr in CD4^+^ cells in the MLNs and spleen in mice detected by flow cytometry. ^a^ *p* < 0.05 compared to the control group. ^b^ *p* < 0.05 compared to the model group. ^c^ *p* > 0.05 compared to the model group. ^d^ *p* > 0.05 compared to the control group. *n* ≥ 9 per group.

Finally, we detected the percentages of Tfh and Tfr in CD4^+^ cells in the MLNs and spleen by flow cytometry (Figures 9, 10). The results showed that percentages of CD4^+^CXCR5^+^PD-1^+^ Tfh and Tfh/Tfr ratios in the MLNs and spleen of colitis mice increased significantly compared to those of normal mice (*p* < 0.05), while no statistical difference was found in CD4^+^CXCR5^+^Foxp3^+^ Tfr (*p* > 0.05) (Figure 11). Compared to the model group, the percentages of Tfh and Tfh/Tfr ratios in the MLNs and spleen in BM-MSCs and Tnfaip6 groups were significantly lower in the MSCs and Tnfaip6 groups (*p <* 0.05), while the percentages of Tfr were significantly higher (*p <* 0.05) (Figure 11). However, the Tfh/Tfr polarization effect of BM-MSCs was abrogated when Tnfaip6 was knocked down (Figure 11).

## Discussion

Clinical data support MSC transplantation as a promising approach for the treatment of IBD (Panes et al., 2016; Panes et al., 2018; Cheng et al., 2019; Shi et al., 2019), and the route of cell infusion may influence the therapeutic effect. Three injection methods commonly used in animal experiments for MSC transplantation to treat IBD were intravenous injection, intraperitoneal injection, and local injection through lesions. However, the optimal injection route for MSC transplantation in IBD animal experiments is yet controversial, and most studies have shown that intraperitoneal injection is better than intravenous injection and local lesion injection (Castelo-Branco et al., 2012; Gonçalves et al., 2014; Sala et al., 2015; Wang et al., 2016).

In this study, MSCs were injected intraperitoneal in the mouse model of IBD. The results showed that intraperitoneal injection of MSCs increased the survival rate and improved the symptoms of TNBS-induced enteritis mice, including reduced food and water intake, weight loss, rough hair, laziness, diarrhea, hematochezia, and other symptoms, and reduced the DAI score and promoted the recovery of colitis. Consistent with these findings, the results suggested that transplanting MSCs *via* intraperitoneal injection is effective in treating TNBS-induced colitis.

In addition, MSC transplantation has shown a good prospect in the treatment of IBD; however, the mechanisms underlying the immune regulation of MSCs on IBD are not yet elucidated. Previous studies have shown that MSCs ameliorate colitis by downregulating the pro-inflammatory cytokines TNF-α, IL-1β, IL-2, IL-6, IL-17, and IL-23; upregulating IL-4, IL-10, and other anti-inflammatory cytokines; and regulating the imbalance of T cell subsets, such as Th 1 cells, Th2 cells, Th17 cells, and Tregs (Ghannam et al., 2010; Chen et al., 2013; Yan et al., 2014; Gao et al., 2016; Fu et al., 2018; Alves et al., 2019). In addition, IBD is also closely related to the imbalance of Tfh/Tfr. Wang et al. (2017) demonstrated that circulating Tfh and serum IL-21 concentration increased, while Tfr decreased in UC patients, and the Mayo score, C-reactive protein, and the erythrocyte sedimentation rate were positively correlated with the Tfh/Tfr ratio and serum IL-21 concentration and negatively correlated with serum IL-10 concentration. Another study also found that Tfr was dysregulated in UC patients and the level of dysregulation was associated with the severity of UC (Yao et al., 2021). Our previous study found that Tfh and Tfr in the intestinal germinal center had abnormal regulation and differentiation in both UC and CD patients (Yang et al., 2020). These results indicated that the imbalance of Tfh/Tfr might shift the immune balance from tolerance to a responsive state, contributing to the pathogenesis of IBD. However, whether MSCs can modulate Tfh/Tfr in IBD remains unclear.

Next, we focused on Tfh and Tfr alterations in colitis mice after MSC transplantation. We found that the expression level of BCL-6, the main transcription factor of Tfh, and the effector cytokine IL-21 in the colon tissue of the IBD model mice were higher than those in the normal mice. Tfh infiltration and the Tfh/Tfr ratio in the MLNs and spleen were significantly increased accompanied by a significant increase in serum pro-inflammatory factors, such as IL-21 and TNF-α concentrations, consistent with our previous results in IBD patients (Yang et al., 2020). After MSC transplantation, the levels of BCL-6 and IL-21 in the colon tissue and the infiltration of Tfh in the MLNs and spleen were decreased, accompanied by a decline in serum IL-21 and TNF-α, while Foxp3, the main transcription factor of Tfr, and the effector cytokine IL-10 in colon tissue significantly increased, Tfr in the MLNs and spleen also increased significantly, the ratio of Tfh/Tfr decreased, and the serum IL-10 concentration increased significantly. These results suggested that MSCs improve colitis by inhibiting Tfh-mediated immune response and promoting Tfr differentiation, regulating the imbalance of the Tfh/Tfr ratio.

Previously, the studies attributed the therapeutic effect of MSCs to their migration, localization, and differentiation to target tissues to replace the damaged cells (Komori et al., 2005; Hayashi et al., 2008). While many stem cell transplantation studies have shown that the number of MSCs reaching target tissues is limited (Sala et al., 2015), the biologically active substances secreted by MSCs and cell-free–conditioned medium showed similar functions in various diseases (Monsel et al., 2016; Mardpour et al., 2019; Zhou et al., 2019). These results suggested that the immunomodulatory effects of MSCs are not related to the tissue homing and differentiation ability but depends on the paracrine effect. Recent studies reported that MSCs exert immunomodulatory effects by secreting soluble multifunctional molecules, including prostaglandin E2, indoleamine-pyrrole 2,3-dioxygenase, transforming growth factor-β, and Tnfaip6 (Li and Hua 2017; Zhou et al., 2019; Song et al., 2020). Among these, Tnfaip6 plays a key role in several immune diseases, such as type 1 diabetes, arthritis, and IBD (Mindrescu et al., 2000; Kota et al., 2013; Sala et al., 2015; Song et al., 2018). Tnfaip6 can be upregulated by MSCs under the stimulation of inflammatory signals, and then mediate a variety of immune regulatory functions of MSCs by binding to a large array of ligands (Day and Milner 2019). Subsequent studies found that in IBD animal models, Tnfaip6 secreted by MSCs reduced DAI and the intestinal histopathologic score, decreased the level of pro-inflammation including IL-6, TNF-α, and IFN-γ, increased the level of anti-inflammatory molecules such as IL-10 and Foxp3, and promoted the proliferation of Tregs and M2 macrophages *via* the immunosuppressive function (Sala et al., 2015; Song et al., 2018).

Then, we explored the role of Tnfaip6 in the regulation of Tfh/Tfr in MSC transplantation in IBD model mice. The serum Tnfaip6 concentration of colitis mice was higher than that of the normal mice. This phenomenon was consistent with the findings of Yu et al. (2016) in IBD patients. After MSC transplantation, the serum Tnfaip6 concentration was increased further, suggesting that in the treatment of IBD by MSC transplantation, Tnfaip6 may be closely related to the outcome of IBD. The exogenous injection of Tnfaip6 replicated the efficacy both clinically and histologically. In terms of immune regulation, intraperitoneal injection of recombinant mouse Tnfaip6 also reduced the infiltration of Tfh, increased Tfr, adjusted the imbalance of Tfh/Tfr ratios in the MLNs and spleen, and reduced inflammation, while MSCs failed to attenuate colitis or regulate the imbalance of the Tfh/Tfr ratio when Tnfaip6 was knocked down. These results supported the crucial role of Tnfaip6 in MSC therapeutic and immunomodulatory effects.

In summary, this study provides new evidence on the immune regulation of MSCs to modulate Th cells in IBD, inhibiting Tfh differentiation, promoting the expansion of Tfr, improving the imbalance of the Tfh/Tfr ratio, and reducing the inflammatory response. Our findings also revealed that this immunoregulation of MSCs is mediated by the release of Tnfaip6. This study might provide a new theoretical basis for further research on the pathogenesis and clinical treatment of IBD. Thus, altered Tfh/Tfr imbalance may be a potential therapeutic target for IBD, while Tnfaip6 might also be expected to become an alternative treatment option for MSC transplantation in IBD in the future.

## Data Availability

The original contributions presented in the study are included in the article; further inquiries can be directed to the corresponding author.
